# New Mediastinal and Hilar Lymphadenopathy After Adjuvant Pembrolizumab in a Patient With Stage III Clear Cell Renal Cell Carcinoma

**DOI:** 10.7759/cureus.76514

**Published:** 2024-12-28

**Authors:** Matthew Lee, Kim Styrvoky, Qi Cai, Phillip Taboada, Remington Hoyer, Zane Gray, Nicholas Levonyak, Luis De Las Casas, Jue Wang

**Affiliations:** 1 Medical School, University of Texas Southwestern Medical Center, Dallas, USA; 2 Internal Medicine, University of Texas Southwestern Medical Center, Dallas, USA; 3 Pathology, University of Texas Southwestern Medical Center, Dallas, USA; 4 Internal Medicine/Oncology, University of Texas Southwestern Medical Center, Dallas, USA

**Keywords:** diagnostic challenges, immune checkpoint inhibitors (icis), immune-related adverse events (iraes), mediastinal lymphadenopathy, multidisciplinary care, renal cell carcinoma (rcc), sarcoidosis-like reaction (slr)

## Abstract

The advent of immune checkpoint inhibitors (ICIs) has transformed the management of advanced and high-risk renal cell carcinoma (RCC). In the adjuvant setting, ICIs, such as pembrolizumab, aim to reduce the risk of recurrence following potentially curative nephrectomy. However, this therapeutic approach introduces unique challenges, particularly related to immune-related adverse events (irAEs). The sarcoidosis-like reaction is a particularly rare immune-related adverse event that can be a diagnostic challenge because of its broad clinical symptoms and potential to mimic metastasis. We present a case of a 50-year-old patient who developed mediastinal and hilar lymphadenopathy during adjuvant pembrolizumab therapy following nephrectomy for stage III RCC, which was initially suspected to be metastatic renal cell carcinoma. Endobronchial ultrasound (EBUS) biopsy revealed non-caseating granulomas without malignancy, leading to a diagnosis of pembrolizumab-induced sarcoidosis-like reaction (SLR). The patient was treated with corticosteroids due to progressive fatigue, leading to a complete resolution of his constitutive symptoms and mediastinal lymphadenopathy. This study highlights diagnostic and therapeutic challenges, the importance of distinguishing irAEs from cancer progression, and strategies to avoid cognitive bias in clinical decision making.

## Introduction

Adjuvant immunotherapy for high-risk renal cell carcinoma (RCC) post-nephrectomy has shown promise in clinical trials, particularly with pembrolizumab. The KEYNOTE-564 trial demonstrated a significant improvement in disease-free survival (DFS) and overall survival (OS) with adjuvant pembrolizumab compared to placebo [1,2] However, the use of immune checkpoint inhibitors (ICIs) in this setting is associated with immune-related adverse events irAEs [1,2]. ICI-induced sarcoidosis-like reaction (SLR) is an exceptionally uncommon irAE, virtually indistinguishable from sarcoidosis, but with a temporal association with the initiation of the ICI [3,4]. Both conditions can be challenging to diagnose as they have broad clinical symptoms [3]. The diagnosis of sarcoidosis, and by extension, SLR, broadly relies on the following three key components: (1) the presence of compatible clinical symptoms, (2) non-necrotizing granulomas, and (3) the exclusion of alternative causes of disease [5].

ICI-induced SLR poses a significant diagnostic challenge [6], with the lungs involved in 60% of cases [7,8]. This can mimic metastasis, as the lungs are the most common site of metastasis for RCC [9]. The overlap in clinical presentation and imaging findings between SLR and metastasis can lead to diagnostic uncertainty, especially since the lungs are frequently involved in both conditions [7-9]. We present a case of a 50-year-old firefighter who developed mediastinal and hilar lymphadenopathy during surveillance imaging after completing one year of adjuvant pembrolizumab following nephrectomy for stage III clear cell RCC. This study underscores the importance of distinguishing irAEs from cancer recurrence, navigating therapeutic decisions, and recognizing the need for a systematic approach to avoid cognitive biases in oncologic care.

## Case presentation

A 50-year-old Hispanic male with a history of obstructive sleep apnea and hypertension underwent an ultrasound, which revealed a left renal mass. After the ultrasound, the patient underwent a left partial nephrectomy, with pathology confirming a 2.9 cm clear cell RCC with tumor extension into the perinephric fat (WHO/International Society of Urological Pathology {ISUP} grade 3, pT3a, cN0, CM0). Post-operatively, the patient started pembrolizumab as adjuvant therapy.

After six cycles of pembrolizumab, imaging revealed several small 1-2 mm lung nodules. However, the patient was asymptomatic. Six months later, a chest CT scan revealed a left lower lobe nodule that had grown from 2 mm to 3-4 mm. The patient reported a minor cough and fatigue, but the lesion was too small for intervention. A fluorodeoxyglucose (FDG) PET/CT scan was performed to restage his cancer, which identified several FDG-avid mediastinal and hilar lymph nodes. These findings raised suspicion for a differential diagnosis of infection, metastatic disease, or autoimmune conditions such as sarcoidosis. Given these findings, the patient’s last cycle of pembrolizumab was held, and he underwent extensive workup including serological, immunological workup, and microbiological studies at the medical oncology office (Table 1).

**Table 1:** Pertinent laboratory results.

Laboratory parameter	Reference range	Values
Soluble interleukin 2 receptor (pg/mL)	175.3-858.2	1524.6
Angiotensin-converting enzyme (U/L)	16-85	6
C-reactive protein (mg/dL)	<0.5	<0.3
Interleukin 6 (pg/mL)	≤2.0	<2.0
Calcium (mg/dL)	8.4-10.2	10.6

**Figure 1:**
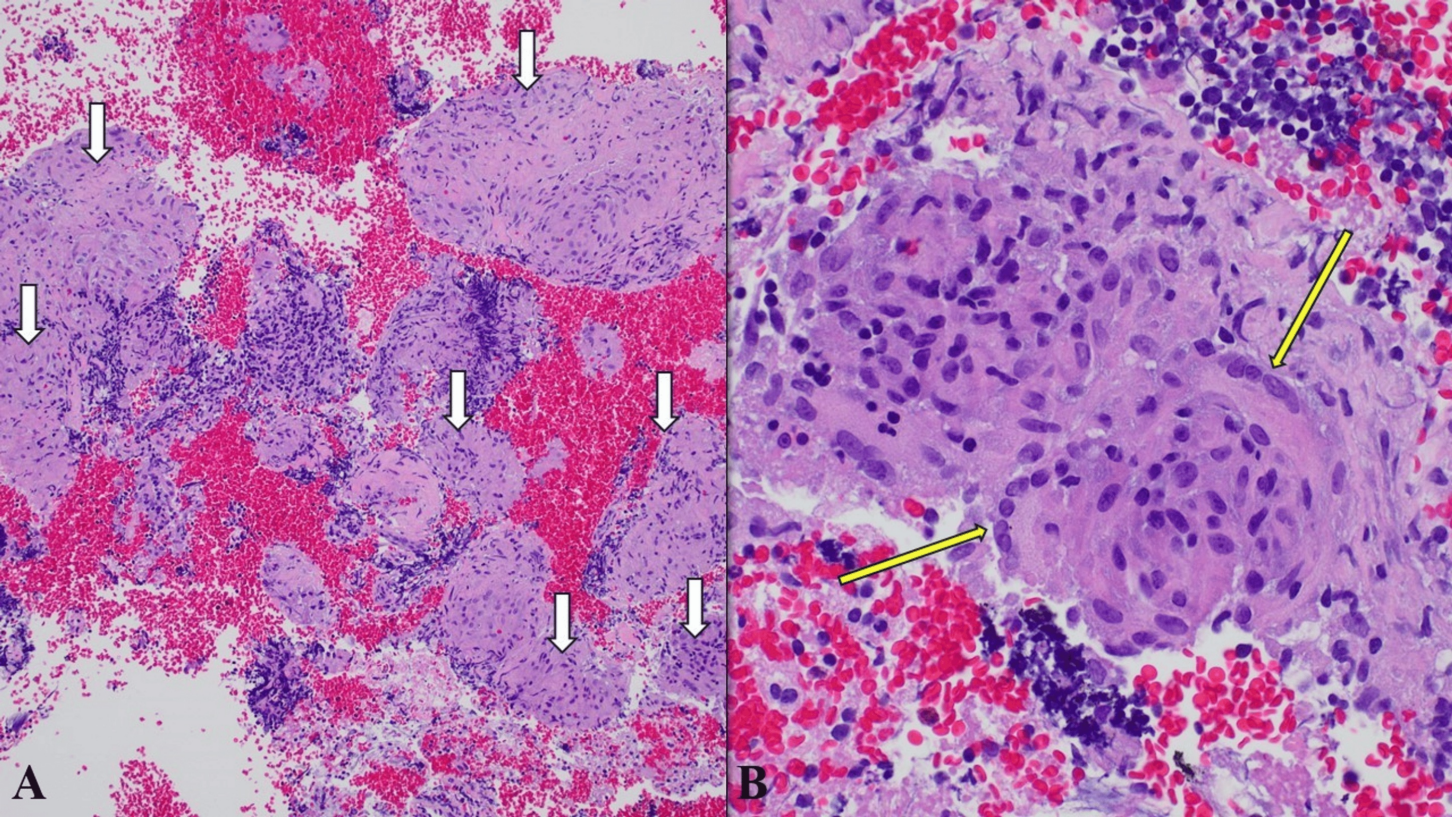
Histopathological characteristics of sarcoidosis-like reaction. (A) Tissue section: low power view showing numerous discrete, non-necrotizing granulomas with compact epithelioid histiocytes (white arrows). Hematoxylin-eosin stain, original magnification 100x. (B) Tissue section: high power view highlighting multinucleated giant cells as part of the granuloma (yellow arrows). Hematoxylin-eosin stain, original magnification 400x.

He was referred to pulmonology and underwent pulmonary function tests and a workup for fungal infections. The pulmonary function tests revealed air trapping and a moderately reduced diffusion lung capacity (Table 2). The fungal workup had negative findings for Blastomyces antibody, Histoplasma mycelia and yeast antibody, Aspergillus antibody, and Fungitell (1,3)-beta-D-glucan. The patient then underwent an endobronchial ultrasound (EBUS)-guided transbronchial fine needle aspiration biopsy of the lymph nodes. The biopsy showed non-necrotizing granulomas (Figure 1, panels A and B).

**Table 2:** Pulmonary function test results. FEV1: forced expiratory volume; FVC: forced vital capacity

Laboratory parameter	Reference range	Values
Diffusion lung capacity (mL/min/mmHg)	20.00-33.74	15.47
Total lung capacity (L)	5.26-7.80	6.07
Forced vital capacity (L)	3.41-5.39	4.05
Forced expiratory volume (L)	2.68-4.22	3.09
FEV1/FVC (%)	67.81-88.85	76.42

**Figure 2:**
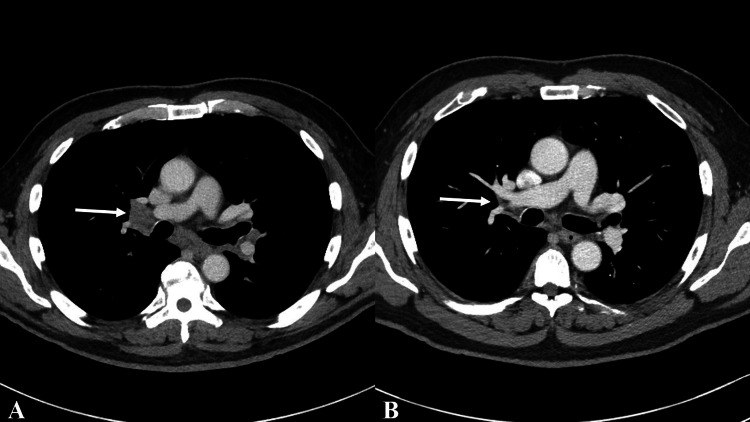
CT images showing suprahilar (arrow) and subcarinal lymphadenopathy (A), with subsequent improvement six weeks after steroid taper (B).

Based on the collective findings from clinical evaluation, laboratory tests, imaging studies, biopsy results, microbiological cultures, and histopathological examination, as well as the temporal relationship between the onset of symptoms and ICI therapy, a diagnosis of ICI-induced SLR was established. At that time, the patient was still relatively asymptomatic. Further immunotherapy was discontinued. Two months later a chest CT with IV contrast demonstrated mediastinal and bilateral hilar lymphadenopathy, with some hilar lymph nodes appearing to slightly increase in size (Figure 2, panel A).

The patient reported profound fatigue to the point he struggled to get out of his bed. He also complained of cough, itching, decreased appetite, and weight loss. Due to his symptoms, the patient began a four-week prednisone taper starting at 40 mg daily and reduced by 10 mg per week. Remarkably, the patient reported a tremendous improvement in energy and appetite by the second week of the prednisone taper. A follow-up chest CT scan six weeks after completion of the steroid taper revealed no adenopathy in the chest by CT size criteria (Figure 2, panel B). At the last follow-up, three years after the initial RCC diagnosis, the patient remains free of any clinical evidence of RCC recurrence or SLR and has successfully returned to work as a firefighter.

## Discussion

The goal of adjuvant therapy in high-risk RCC patients post-nephrectomy is to reduce the risk of recurrence and improve disease-free survival (DFS) and OS [9]. Unlike advanced-stage disease, where the primary focus is on disease control, the adjuvant setting requires balancing the management of irAEs with preserving the patient’s disease-free status and quality of life. In the setting of high-risk RCC, new findings such as mediastinal and hilar lymphadenopathy often raise immediate concerns about cancer progression and can influence decisions to escalate therapy for presumed metastatic disease. The aggressive nature of high-risk RCC makes recurrence or metastasis a likely consideration, and clinicians may feel compelled to initiate systemic therapy without additional diagnostic confirmation.

SLR is a rare irAE that may mimic cancer recurrence, compounding the complexity of diagnosis and management in this context [6]. SLR is a diagnosis of exclusion, requiring thorough consideration of differential diagnoses, including metastatic disease, infections, and other autoimmune conditions. The workup includes imaging, laboratory evaluation with serology, microbiological testing, and biomarker analysis, such as C-reactive protein (CRP), erythrocyte sedimentation rate (ESR), angiotensin-converting enzyme (ACE), and soluble IL-2 receptor (sIL-2R), and a biopsy (e.g., endobronchial ultrasound {EBUS}-guided transbronchial needle aspiration {TBNA}) to confirm non-caseating granulomas and exclude other etiologies. Management is guided by the severity of the irAE; mild cases may be monitored, while moderate to severe cases require holding or permanently stopping ICIs and initiating corticosteroids or immunosuppressive therapy.

Our case demonstrates the diagnostic challenges of ICI-induced SLR and the importance of multidisciplinary care. The patient’s extensive workup revealed both typical and atypical findings of SLR. Firstly, his soluble sIL-2R levels were significantly increased at 1524.6 pg/mL (normal range: 175.3-858.2 pg/mL). Elevated sIL-2R levels suggest a heightened immune response and are often a valuable biomarker for diagnosis and monitoring disease severity in sarcoidosis [10,11]. Higher sIL-2R levels are also associated with more extensive pulmonary involvement, offering a useful clue to the patient’s diagnosis of ICI-induced SLR [11]. Conversely, our patient’s ACE levels were unexpectedly low at 6 U/L (normal range: 16-85 U/L), deviating from the usual elevation in sarcoidosis [5,12]. However, ACE levels have a poor sensitivity for detecting sarcoidosis, so this result did not exclude ICI-induced SLR from the differential diagnosis [12]. Both Shrateh et al. and Katagiri et al. reported cases of ICI-induced SLR with elevated ACE levels in metastatic RCC patients [3,10]. This underscores the heterogeneity of ICI-induced SLR, necessitating thorough clinical investigation. Since sIL-2R is more sensitive than ACE for detecting sarcoidosis, it should be used to help differentiate ICI-induced SLR from other causes of inflammation and to monitor disease progress in patients undergoing ICI therapy.

In addition to these laboratory findings, the patient underwent a fungal workup with pulmonology to preemptively exclude fungal causes of pulmonary granulomas, should they be identified upon biopsy. One retrospective study found that 25% of pulmonary granulomas were caused by fungal infections [13]. Because SLR is a diagnosis of exclusion, this workup was essential to narrow the differential diagnosis. Despite suggestive imaging and laboratory findings for SLR, a definitive diagnosis requires pathological confirmation [6,14]. In the present case, the biopsy revealed non-necrotizing granulomas with no sign of metastasis, which supported the diagnosis of ICI-induced SLR. This confirmation was particularly important in our patient to rule out metastatic RCC, given that the lungs are the most common site of metastasis in RCC [8,9]. In contrast, biopsy was not feasible for the RCC patients with ICI-induced SLR reported by Katagiri et al. and Purcell et al.; instead, a presumed diagnosis of SLR was made based on supportive clinical markers, such as elevated ACE levels, a temporal relationship with ICI treatment, and radiographic findings compatible with SLR [10,15]. However, pathologic examination is the gold standard for diagnosing SLR and should be pursued when feasible, particularly to rule out metastatic disease. After an extensive literature review, we identified an additional five cases of ICI-induced SLR in RCC patients (Table 3).

**Table 3:** Reported cases of ICI-induced sarcoidosis-like reaction in RCC patients. ICIs: immune checkpoint inhibitors; RCC: renal cell carcinoma; SLR: sarcoidosis-like reaction

Studies	Age (year)/sex	mRCC (Y/N)	ICI used	Time of onset (after ICI)	Affected organs	Biopsy (Y/N)	Treatment	SLR outcome	Symptoms
Present case (2024)	50/M	N	Pembrolizumab	5.5 Months	Lungs	Y	Stop ICI + prednisone	Resolution	Fatigue, loss of appetite, cough, itching, weight loss
Shrateh et al. (2024) [3]	69/M	Y	Pembrolizumab	12 Months	Lungs	Y	Stop ICI	Improvement	Cough
Charkviani et al. (2023) [16]	60/M	Y	Nivolumab + ipilimumab	2.5 Months	Kidney	Y	Stop ICI + prednisone	Resolution	Fatigue, loss of appetite, decreased urine output
Purcell et al. (2022) [15]	50/M	Y	Nivolumab + ipilimumab	N/A	Lungs	N	Stop ICI	Resolution	Dry cough
Katagiri et al. (2022) [10]	58/M	Y	Nivolumab + ipilimumab	2.25 Months	Lungs	N	Stop ICI	Resolution	Asymptomatic
Zhang et al. (2017) [17]	64/F	Y	Nivolumab	10 Months	Lungs	Y	N/A	N/A	N/A

Notably, four of the five cases had pulmonary SLR and all five cases had metastatic RCC [3,10,15-17]. Beyond RCC, the incidence of ICI-induced SLR across various cancers ranges from 0.2% to 5.0% [7,18,19]. Despite its rarity, there is a need for heightened awareness of ICI-induced SLR as ICIs become increasingly used in cancer treatment.

This study reported a case of SLR in a high-risk RCC patient receiving pembrolizumab after nephrectomy. This distinction is significant, as the diagnostic and therapeutic considerations in the adjuvant setting differ from those in metastatic disease, where the likelihood of cancer progression is inherently high. This case broadens our understanding of the spectrum of irAEs and underscores the importance of vigilance for such reactions, regardless of the cancer stage or treatment intent. Finally, we highlight the importance of considering ICI-induced SLR in the differential diagnosis of RCC patients with pulmonary manifestations and the critical role of pathology in confirming the diagnosis and ruling out metastasis. The present case exemplifies the critical need to recognize and mitigate cognitive biases in clinical practice, particularly anchoring and confirmation biases. Anchoring bias may lead clinicians to fixate on the most obvious diagnosis - in this scenario, cancer recurrence - based on the patient's history. Confirmation bias might drive the interpretation of diagnostic tests to support this initial assumption, potentially overlooking alternative diagnoses. To avoid these pitfalls, a systematic and open-minded approach is essential. Clinicians should maintain a broad differential diagnosis when new symptoms or findings arise, even in the context of a known malignancy. Incorporating multidisciplinary perspectives, including oncology, pulmonology, radiology, and pathology, enriches the diagnostic process. Regular tumor board discussions and consultations can challenge assumptions and promote consideration of less common but significant conditions like pembrolizumab-induced SLR.

Making the distinction between ICI-induced SLR and metastatic RCC is difficult but crucial because both conditions require vastly different treatments. For metastatic RCC, the standard of care includes ICI-based combination therapy [20]. However, in a patient with an irAE such as SLR, additional ICI therapy could exacerbate symptoms. The treatment of SLR involves the discontinuation of ICI therapy, with or without the initiation of corticosteroids [7]. Our initial management approach was tailored to the patient’s clinical presentation. Treatment with corticosteroids was deemed unnecessary since the patient’s symptoms were minor and pembrolizumab had already been held. Notably, three of the identified cases of ICI-induced SLR in RCC patients were managed with the discontinuation of the ICI without additional treatment (Table 3). However, our patient’s condition began to deteriorate with worsening symptoms and increased lymphadenopathy, so he was treated with a prednisone taper. In a broader review of multiple irAEs, Le Burel et al. found that corticosteroids were used in 87% of cases to treat these irAEs [19]. Our patient's rapid and lasting resolution of SLR with prednisone aligns with existing literature and reinforces the efficacy of corticosteroids in managing irAEs such as SLR.

## Conclusions

This case highlights the diagnostic and therapeutic complexities of managing irAEs, particularly pembrolizumab-induced SLR. In the setting of high-risk RCC, new findings such as mediastinal and hilar lymphadenopathy often prompt concerns about cancer progression and can influence decisions to escalate therapy for presumed metastatic disease. We underscore the importance of integrating histological evaluation and a multidisciplinary approach into the decision-making process, ensuring that therapeutic strategies are guided by accurate diagnoses rather than assumptions based on imaging findings alone.
